# A new platform for global health research and policy exchange and communication

**DOI:** 10.1186/s41256-016-0001-z

**Published:** 2016-06-15

**Authors:** Xinguang Chen

**Affiliations:** 1 Global Health Institute, Wuhan University, Wuhan, China; 2 Department of Epidemiology, University of Florida, Gainesville, USA

## Abstract

Peer-reviewed publications are essential to promote the development of global health sciences. Wuhan University Global Health Institute, in partnership with BioMed Central, launches *Global Health Research and Policy* (*GHRP*) as an open access journal to meet the needs of all authors and readers who are interested in global health. Our publication priorities include both empirical and implementation research, research methodologies, and global health education, as well as opinions and discussion over the concept of global health sciences and its development in the future.

## Editorial

On behalf of Dr. Youmei Feng and Dr. Zongfu Mao and our Editorial Office, I am very pleased to present a new scholarly open access journal, *Global Health Research and Policy*. This journal is a timely addition to the literature, providing an exciting platform to meet the growing need of all readers wishing to catch up with new developments in global health, eager to know the current status and future trends in global health research and policies, and looking for new opportunities within global health. The journal will also serve as an information source for those who want to expand their network connections with researchers in the field of global health, and those who are looking for additional education and training, hot research topics, innovative research methodologies, and professional careers in the field of global health.

Dear authors, our journal is created for you to publish your work. We highly value your contributions to our journal. The innovation of your work and the vast reach of our journal through BioMed Central make it an ideal place to promptly realize the impact of your scientific research, while promoting the growth of our journal—a win and win choice. Please keep our journal as one of your top choices for manuscript publication. We will try our best to provide the most efficient services to review your papers, to obtain thoughtful and constructive critiques from the reviewers, and to make prompt decisions regarding publication of your work. We also welcome your comments and suggestions to improve our journal and to better capture the development in global health research and policy.

Global health, often dubbed as global health sciences, is a totally new branch of science within the medical and public health field. Different from many well-established traditional disciplines, such as international health, public health, biostatistics, epidemiology, environmental health, maternal and child health, nutrition and food hygiene, global health is still in its early and developmental stages. Any medical and health issue can be considered global health if it presents globally, or possesses a global impact, or can better be solved only if a large-scale inter-international or a global strategy is taken. Global health can be used as open guidance to help understand challenging medical and health issues, such as HIV/AIDS and other infectious diseases, the growing epidemic of overweight and obesity, large scale environmental pollution, global warming, differences in access to healthcare, and regional/global health inequality.

A number of disciplines have become established within global health sciences. Typical examples include global child health, global aging, global pharmacology, global HIV/AIDS research, global oral health, global eye health, and global epidemiology. Although focusing on different areas, a characteristic common to all these sub-disciplines is the utilization of a global perspective as the guidance. As an open platform for all researchers, we welcome all types of research findings from you as long as the work is guided by a global perspective or can help others to tackle medical and health issues with a global perspective. We are particularly interested in papers reporting empirical research and implementation of global health programs, new methodologies for global health research, global health education, opinions and discussion over the concept of global health sciences and its development in the future.

The launching of *Global Health Research and Policy* is not possible without many people’s contribution. I am the person who first proposed the idea in 2013 to publish a web-based journal as a flagship to support the development of global health when I participated in a seminar held by Dean Mao in the newly established Global Health Institute (GHI) at Wuhan University. With a collective effort of leaders from the Health and Family Planning Commission of China and Wuhan University, and a group of experts within and outside of China, Wuhan GHI launched a trial version, entitled *Journal of Global Health* in 2014, which published three issues in printed hardcopies, a total of 34 papers. Now, we are pleased to launch *Global Health Research and Policy* as an online open access journal in partnership with BioMed Central.

It is our goal and expectation that the fine collection of all articles published in this journal will be a valuable source for both readers and researchers to promote the growth and development of global health into a vibrant branch of new medical and health science.

